# Characteristics of patients with perinatal depression in UK primary care settings

**DOI:** 10.1192/j.eurpsy.2025.475

**Published:** 2025-08-26

**Authors:** C. Gillis, S. Chen, C. Mak, E. Tworkoski, L. Li, S. Eaton, C. Green, N. Maserejian, M. Koch

**Affiliations:** 1 Biogen, Cambridge, United States; 2 Biogen, Maidenhead, United Kingdom

## Abstract

**Introduction:**

Perinatal depression (PND) is a debilitating mood disorder that occurs during or following pregnancy. Information regarding characteristics associated with PND can aid in better understanding the disease.

**Objectives:**

Examine patient characteristics and comorbidities of PND patients and matched non-PND controls in a UK electronic health record (EHR) database.

**Methods:**

Women aged 12-55y with a delivery record (livebirth, stillbirth, mixed birth) during Jan 2017-Dec 2021 were selected from the Clinical Practice Research Datalink (CPRD) GOLD primary care database via a pregnancy algorithm. PND was ascertained using read codes during pregnancy or one year after the end of pregnancy date (EPD). Two ways to define PND were explored: 1) restricted definition (PND-r) where only PND codes were included; 2) broad definition (PND-b) including both PND and 34 other depression codes. For each PND case, two controls were matched on key patient variables such as age, depression history, and pregnancy outcome. Patients were required to have at least 12 months (mo) of continuous enrollment prior to and post EPD. Patient characteristics and select psychiatric and pregnancy-related comorbidities were compared, and prevalence rate ratios (PRR) and 95% Wald confidence intervals (CI) were calculated comparing cases and controls.

**Results:**

The study included 2,214 cases of PND-r and 4,718 cases of PND-b, and their respective non-PND matched controls. PND-r and PND-b cases differed in timing of diagnosis. Of the PND-r cases, 6% were diagnosed during pregnancy and 47% in the first 3mo following delivery; whereas the distribution in the PND-b was, respectively, 18% and 30%. The mean age of the PND-r and PND-b cohorts was consistent (29y, SD=6). A prior recorded history of depression was present in 36% of PND-r cases and 43% of PND-b cases. Approximately 55% of PND-r cases and 65% of PND-b cases had a history of antidepressant use prior to last menstrual period.

During the 12mo pre-EPD, PND-r cases had higher prevalence of hyperemesis gravidarum (PRR=1.5, 95% CI 1.3-1.8), gestational diabetes (PRR=1.4, 95% CI 1.1-1.7), and anxiety/panic disorders (PRR=1.8, 95% CI 1.3-2.3) compared to controls. During the 12mo post-EPD, PND-r cases had higher prevalence of comorbid anxiety/panic disorders (PRR=3.8, 95% CI 3.0-4.9) as well as post-traumatic stress disorder (PRR=3.1, 95% CI 1.5-6.1) compared to controls. Results were similar in the PPD-b analyses, but with higher PRR of anxiety/panic disorders (post-EPD PRR: 11.2, 95% CI 9.7-12.9).

**Conclusions:**

80% of PND-r and 71% of PND-b cases were diagnosed during pregnancy or in the first 6mo following EPD. Cases had higher prevalence of certain pregnancy and psychiatric conditions compared to controls, with comorbid anxiety/panic disorder estimates higher using the PND-b definition. Future research should consider comparing results using both broad and restricted definitions of PND in EHRs.

**Disclosure of Interest:**

C. Gillis Shareolder of: Biogen, Employee of: Biogen, S. Chen Shareolder of: Biogen, Employee of: Biogen, C. Mak Shareolder of: Biogen, Employee of: Biogen, E. Tworkoski Shareolder of: Biogen, Employee of: Biogen, L. Li Shareolder of: Biogen, Employee of: Biogen, S. Eaton Shareolder of: Biogen, Employee of: Biogen, C. Green Shareolder of: Biogen, Employee of: Biogen, N. Maserejian Shareolder of: Biogen, Employee of: Biogen, M. Koch Shareolder of: Biogen, Employee of: Biogen.

